# Targeting the DNA damage response in cancer

**DOI:** 10.1002/mco2.788

**Published:** 2024-10-31

**Authors:** Guffanti Federica, Chiappa Michela, Damia Giovanna

**Affiliations:** ^1^ Laboratory of Preclinical Gynecological Oncology Department of Experimental Oncology Istituto di Ricerche Farmacologiche Mario Negri IRCCS Milan Italy

**Keywords:** ATM, ATR, Chk1, DNA damage response, Polθ, solid tumors, Wee1

## Abstract

DNA damage response (DDR) pathway is the coordinated cellular network dealing with the identification, signaling, and repair of DNA damage. It tightly regulates cell cycle progression and promotes DNA repair to minimize DNA damage to daughter cells. Key proteins involved in DDR are frequently mutated/inactivated in human cancers and promote genomic instability, a recognized hallmark of cancer. Besides being an intrinsic property of tumors, DDR also represents a unique therapeutic opportunity. Indeed, inhibition of DDR is expected to delay repair, causing persistent unrepaired breaks, to interfere with cell cycle progression, and to sensitize cancer cells to several DNA‐damaging agents, such as radiotherapy and chemotherapy. In addition, DDR defects in cancer cells have been shown to render these cells more dependent on the remaining pathways, which could be targeted very specifically (synthetic lethal approach). Research over the past two decades has led to the synthesis and testing of hundreds of small inhibitors against key DDR proteins, some of which have shown antitumor activity in human cancers. In parallel, the search for synthetic lethality interaction is broadening the use of DDR inhibitors. In this review, we discuss the state‐of‐art of ataxia‐telangiectasia mutated, ataxia‐telangiectasia‐and‐Rad3‐related protein, checkpoint kinase 1, Wee1 and Polθ inhibitors, highlighting the results obtained in the ongoing clinical trials both in monotherapy and in combination with chemotherapy and radiotherapy.

## Graphical abstract

DNA damage response pathway can be effectively targeted with different small molecules inhibitors. They can be used in tumors with specific genetic defects (synthetic lethal approach), in combination with chemotherapy and radiotherapy, in combination with immune checkpoints and in the resistant setting.

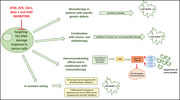

## 1 INTRODUCTION

DNA integrity is important for cell survival. Cells are continually exposed to DNA damage caused by endogenous (reactive oxygen species) and exogenous (ultraviolet [UV] light, irradiation, chemical compounds) factors that must be repaired as quickly as possible to prevent the damage from becoming fixed in the DNA and being passed on to daughter cells.^1^ The cellular coordinated network to cope with DNA lesions is the DNA damage response (DDR) pathway which identifies, signals, and repairs the damage. It tightly regulates cell cycle progression and promotes DNA repair to minimize DNA damage to daughter cells.^2^, ^3^ Too severe damage cannot be repaired and results in cell death. However, unfaithful repair and/or partially repaired DNA damage can have deleterious effects resulting in accumulation of damages leading to genomic instability and cancer development. Defects in genome maintenance and repair have been shown to be advantageous and instrumental for tumor progression and this condition has been annotated as an hallmark of cancer.^4^ The physiological importance of the DDR is underlined by the fact that mutations of DDR genes are frequently found in cancers and that germline mutations of these genes predispose to cancer development.^5^

The unravelling of the molecular mechanisms underlying DDR has led to speculation that its inhibition may open up new therapeutic opportunities in oncology for several reasons. First, anticancer treatments involve the use of radiotherapy (RT) and DNA‐damaging agents, which stress the integrity of the genome and activate the DDR response, the inhibition of which could delay the repair of DNA lesions and lead an enhancement of their antitumor activity. Second, DDR dysfunction, reported in many cancers, has emerged as the Achilles’ heel of tumors. Indeed, defects in DDR have been shown to make cancer cells not only more susceptible to DNA‐damaging agents but also more dependent on the activity of the remaining intact DDR pathways, inhibition of which would lead to cell death by synthetic lethality.^6^, ^7^ All this knowledge has led not only to the development of small molecules that inhibit key proteins involved in DDR, but has also spurred research to identify synthetic lethal interactions among DDR proteins to be exploited therapeutically.

In this review, we will discuss the DDR, its importance for cancer development, and its therapeutic value, with the specific focus on key DDR proteins and their inhibition as a strategy in oncology. In particular, we report on the preclinical development of ataxia‐telangiectasia mutated (ATM), ataxia‐telangiectasia‐and‐Rad3‐related protein (ATR), checkpoint kinase 1 (Chk1), Wee1, and Polθ small molecule inhibitors and, whenever available, the results of their ongoing clinical trials.

## 2 OVERVIEW OF THE DNA DAMAGE RESPONSE

DDR has been evolved to maintain genomic integrity, as DNA is the repository of genomic information and has to be preserved for a proper cell survival and organism maintenance. In recent decades, not only the mechanisms underlying this network have been unraveled, but new protein‒protein interactions, new physiological functions of old proteins, and other catalytic processes are rapidly emerging.^8^

**FIGURE 1:**
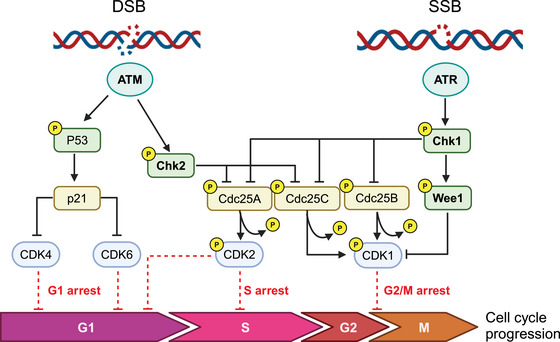
Schematic overview of the DNA damage response (DDR) pathway. The schematic activation of the DDR after single and double DNA strand breaks (SSB and DSB, respectively) is illustrated. As shown, the activation of ataxia‐telangiectasia mutated (ATM) and ataxia‐telangiectasia‐and‐Rad3‐related protein (ATR) lead to the downstream phosphorylation of checkpoint kinase 1/2 (Chk1/2) kinases that will activate downstream proteins leading to cell cycle arrest to facilitate repair. CDK, cyclin‐dependent kinase. The figure has been created by the authors using BioRender.

DDR begins with the recognition of the DNA lesion and the engagement of DNA repair, strictly linked to cell cycle machinery that will be stopped and/or slowed down to allow these processes to take place (Figure 1). Depending on the type of lesion, specific intracellular signaling events are triggered; from alteration of chromatin surrounding the lesion to recruitment of repair proteins, activation of cell cycle checkpoints and of gene expression (both by transcription and/or translation mechanism). Of note, DDR is very rapid because it relies on a cascade of phosphorylation events (more than 900 for a single DDR event^9^, ^10^), making DNA repair the most important cellular energy‐consuming process.^11^

We will briefly summarize the activation of DDR network upon DNA damage, taking into account that this is a multistep process involving upstream sensors able to recognize the damage and transduce the signaling to downstream targets to activate, if possible, repair and simultaneously cause the cell cycle to block and/or slow‐down in order to properly repair the damage.

### 2.1 Upstream sensors and transducers

ATM and ATR are the key mediators of the DDR, with the specific function of orchestrating the cell's response to damage and initiating the cascade of events leading to cell cycle arrest and repair. Both are serine‐threonine kinases, belonging to the phosphoinositide 3‐kinase (PI3K) family, and once activated regulate, by phosphorylation, a high number of interconnected proteins.^9^ Specifically, both these proteins orchestrate the cellular response to DNA double strand breaks (DNA‐DBSs) and replication stress (RS). Chk1 and Chk2 are the major cell cycle checkpoints proteins activated respectively by ATR and ATM and halt the cell cycle progression by inhibiting the cell‐cyclin‐dependent kinases (CDK).^12^, ^13^, ^14^

After damage, the MRN (MRE11—the meiotic recombination 11 homolog 1; RAD50; NBS1—phosphopeptide‐binding Nijmegen breakage syndrome protein 1) complex localizes to the site of DNA damage and activates ATM through interaction with NBS1.^15^, ^16^, ^17^, ^18^ The activation of ATM starts with autophosphorylation at Ser1981 and with the phosphorylation of the three MRN proteins.^19^, ^20^ The ATM‐dependent phosphorylation of the MRN complex results in its conformational change allowing its binding to DNA.^21^ Once activated, ATM phosphorylates downstream proteins including Chk1, Chk2, the p53 protein (TP53 or p53), H2AX, breast cancer type 1 susceptibility protein (BRCA1), and carboxy‐terminal binding protein interacting protein (CtIP).^18^ During G1 phase, the phosphorylation of p53 results in its stabilization and in the transcription of CDKN1A, which encodes the CDK inhibitor p21; Chk2 phosphorylation leads to Cdc25A degradation, a phosphatase required for cyclin E/CDK2 activation; both these events lead to a blockage in the G1/S phase of the cell cycle.^22^, ^23^, ^24^ The activation of 53BP1 during G1 phase favors non‐homologous end joining (NHEJ) repair.^24^ On the contrary, during S/G2 phase, the phosphorylation of CtIP protein by ATM promotes both DNA strand resection necessary for homologous recombination (HR) to occur and removal of proteins required for NHEJ.^22^ ATM signaling leads to G2/M cell cycle arrest through the phosphorylation of Chk2 on Thr68, Ser19, Ser33/35, or Ser50 inducing Chk2 monomers dimerization and autophosphorylation of its kinase domain.^20^ Chk2 phosphorylates, both the Cdc25A and Cdc25C phosphatases, resulting in their inactivation. Cdc25A dephosphorylates CDK2, promoting progression into S phase, while Cdc25C activates CDK1 allowing G2/M progression.^25^ Other substrates of Chk2 determining the G2/M arrest are the dual specificity protein kinase TTK/hMps1, whose mechanism has not yet been fully described^26^; the serine/threonine kinase receptor‐associated protein STRAP, a p53 cofactor that can induce a p53‐dependent G2/M arrest^27^; and the RNA polymerase II‐binding protein Che‐1, whose active isoform is recruited on the promoters of p21 and p53 genes promoting their transcription.^28^ ATM can cause a G1 cell cycle arrest by phosphorylating p53 on Ser15 and Ser20 and its regulatory ubiquitin ligase MDM2 on multiple sites to prevent its ubiquitination and proteasomal degradation leading to p53 stabilization and activation.^29^, ^30^ Recently it has been demonstrated that ATM interacts directly with p53 mRNA. After DNA damage MDM2 and its homolog MDMX compete with ATM for binding the p53 mRNA, enhancing its translation.^31^ Active p53 promotes the transcription of CDKN1A, which encodes the CDK p21 causing G1 cell cycle arrest^32^ and, if the damage is sustained, the transcription of different pro‐apoptotic genes including Puma, Fas‐R, Noxa, BAX, Apaf1, Noxa, and Pidd resulting in apoptotic cell death.^33^ Although ATM can arrest cell cycle both to G2/M and G1/S phase, cell cycle defects observed in ATM‐deficient cells are primarily G1/S checkpoint deficiency.^34^

Mutations in the ATM gene are responsible for a rare autosomal recessive pathology ataxia‐telangiectasia (A‐T), characterized by cerebellar degeneration, ataxia, skin telangiectasia, immune disfunction, and increase cancer incidence.^35^ In addition, A‐T cells are extremely sensitive to ionizing radiation (IR) due a defect in DNA‐DBS repair and displayed chromosome breakage.^36^ ATM mutations are found in many solid tumors (breast, ovarian, colorectal, and prostate) and hematological malignancies; in addition, inactivating mutations of ATM characterize half of mantle cell lymphoma and T‐cell prolymphocytic leukemia patients.^37^

The second mediator of DDR is ATR, that is mainly activated in presence of persistent single‐stranded DNA (ssDNA) structures, common intermediates formed at stalled replication forks, during RS (an alteration of replication fork progression with a reduced replication fidelity leading to the formation of DNA) and during DNA repair activity of nucleotide excision repair (NER) and HR pathways breaks.^6^, ^38^, ^39^, ^40^ ATR recognizes ssDNA filaments by its constitutive partner ATR interacting protein (ATRIP), which directly interacts with replication protein A (RPA) bound to ssDNA filaments, and their interaction elicits ATR activation.^41^, ^42^ Post‐translational modifications are required to coordinate the assembly and functions of Rad17‐replication factor C (RFC) and the complex Rad9‐Rad1‐Hus1 (9‐1‐1) with ATRIP‐ATR to generate a docking site^43^, ^44^ to recruit the final activator of ATR, the topoisomerase binding protein 1 (TOPBP1)^45^ leading to its fully activation.^46^ ATR phosphorylates a wide range of downstream targets, including Chk1, triggering signal cascades both at the site of DNA damage to coordinate the DNA repair activity of HR, NER and Fanconi anemia (FA),^47^, ^48^, ^49^ and more globally to regulate replication forks dynamics during S phase,^50^, ^51^ cell cycle checkpoints,^39^ or elicit apoptosis through p53.^52^

ATR activates both intra‐S and G2/M checkpoints in response to RS and DNA damage by phosphorylating Chk1 on Ser‐317 and Ser‐345, which achieves full activation with autophosphorylation at serine 296.^53^ Similar to Chk2, Chk1 inactivates Cdc25A, leading to a decrease in CDK2 activity in S phase,^54^ and Cdc25B/C causing a G2/M arrest.^55^ Chk1 directly targets and activates WEE1, a serine‒threonine kinase that phosphorylates CDK1 at Tyr15 and inhibits CDK1 kinase activity triggering G2/M arrest^56^, ^57^ ATR/CHK1 axis has a role also in the stabilization of stalled replication forks, where it acts as an intra‐S phase checkpoint, ensuring that activation of late replication origins is blocked and replication fork integrity is maintained when DNA synthesis is inhibited.^58^ ATR is necessary to cope with RS, through the activation of CHK1.^59^ As proliferating tumor cells have high levels of RS, they rely on ATR.^60^

Germline mutations in ATR lead to Seckel syndrome, a rare autosomal recessive disorder characterized by proportional short stature, dysmorphic facial appearance, and mental retardation.^61^ In human tumors, ATM has been reported to be mutated in 1394 (3.0%) of the 46,588 samples analyzed.^62^ Given the essential role of the ATR‐Chk1‐Wee1 axis in the RS response, genomic alterations of this pathway are very low (<3% for ATR and <1% for Chk1 and Wee1).^63^ ATM and ATR have overlapping activities with substantial crosstalk between the two pathways as they share many substrates; however, they are non‐redundant and cannot compensate for the loss of each other.^64^, ^65^

### 2.2 DNA repair pathways

Repair of the damage is therefore a key element in the DDR pathway and cells are equipped with several repair mechanisms that deal with different DNA lesions.^6^, ^66^, ^67^ Typically, these pathways share many proteins and are often cross‐connected. In addition, when one mechanism is deficient, others are upregulated. It has been clearly demonstrated that specific DNA lesions generally activate distinct damage‐sensing and repair pathways. The most common DNA lesions are the ones affecting the single DNA strand either the SSBs in the phosphate backbone or by chemical modification of the DNA bases.^68^ Generally, these lesions are repaired by the base excision repair (BER), nucleotide excision repair (NER) or can eventually be bypassed during DNA replication by translesion synthesis (TLS). The mismatches generated during DNA synthesis are repair by the mismatch repair (MMR),^69^ while the mis‐incorporated ribonucleotides are removed by the ribonuclease H2.^70^ While the SSBs are the most common and easy to repair DNA lesions, the DNA‐DSBs involving both DNA strands, are a great threat to genomic integrity and are far more difficult to be fixed. The two major pathways involved in their repair are the NHEJ and HR pathways.

**FIGURE 2:**
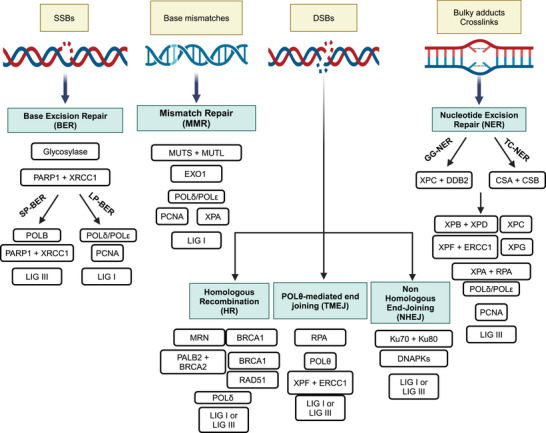
Schematic overview of the main DNA repair pathways. The figure summarizes the main DNA repair pathways. Base excision repair is involved in the repair of single strands, mismatch repair detects and repairs base mismatches resulting during replication, double strand breaks can be repaired by different pathways (homologous recombination, non‐homologous recombination and Polθ‐mediated end joining) with different degree of fidelity and bulky adducts and crosslinks require the nucleotide excision repair, involving more than 20 proteins. See text for a detailed explanation of these pathways. BER, base excision repair; BRCA1, breast cancer protein 1; BRCA2, breast cancer protein 2; DNAPKcs, DNA‐protein kinase catalytic subunit; DSBs, double strand breaks; LIG, ligase; MMR, mismatch repair; MRN complex, MRE11‐RAD50‐NBS1; PALB2, partner and localizer of BRCA2; PARP1, poly‐ADP‐ribose‐polymerase 1; PCNA, proliferating cell nuclear antigen; POL, polymerase; RPA, replication protein A; SSBs, single strand breaks; XPA, xeroderma pigmentosum, complementation group A; XRCC1, X‐ray repair cross‐complementing protein 1. The figure has been created by the authors using BioRender.

We will briefly describe the main cell DNA repair pathways heightening the key players amenable of inhibition. A schematic overview of the repair pathways is illustrated in Figure 2.

#### 2.2.1 Base excision repair

BER is a high fidelity DNA repair system crucial for fixing the insertion of aberrant DNA bases and repairing DNA SSBs caused by internal and/or exogenous sources.^71^ These lesions are highly diffuse and can interfere with DNA replication and transcription process contributing to genomic instability and oncogenesis.^72^, ^73^, ^74^ BER is a multi‐step process involving various enzymes often shared with other repair mechanisms that initially recognizes and excises the incorrect base followed by the re‐synthesis and ligation with the restoration of the original DNA sequence. In particular, specific DNA glycosylases initially identify and cleave the damaged base creating an apurinic or apyrimidinic site^75^, ^76^ and subsequent apurinic/apyrimidinic endonucleases (APE1 or APE2) recognize the abasic sites and generate SSBs. BER then progresses through short‐patch (SP) or long‐patch (LP) sub‐pathways. SP‐BER is active during G1 phase to quickly repair single base damage, while LP‐BER inserts short sequences of nucleotides during G2/S phases and generally requires more time than SP.^77^ Replacement of the damaged base and re‐ligation of the DNA involve binding of poly(ADP‐ribose) polymerase 1 (PARP1), DNA polymerases β (POLB), δ or ε and ligase I or III (LIG3). PAR polymers formed by PARP1 induce auto‐modification and post‐translational changes of PARP1 targets such a X‐ray repair cross‐complementary gene 1 (XRCC1), POLB and on the damaged site, whose negative charge promotes chromatin loosening, and facilitates the recruitment of repair proteins and their access to DNA.^78^ POLB replaces the single damaged nucleotide^79^ and attracts LIG3 with the help of the scaffold proteins XRCC1 in SP, or proliferating cell nuclear antigen (PCNA) in LP BER to promote polymerase‒ligase interaction^80^ and the subsequent sealing of the DNA rupture.^81^

BER proteins have been found dysregulated in different tumors (germline, somatic mutations, and polymorphisms)^82^, ^83^, ^84^ and their targeting has been explored as a therapeutic strategy. A paradigmatic example is the inhibition of PARP1 using small molecules in HR‐deficient tumors harboring *BRCA1/BRCA2* mutations exploiting a synthetic lethality approach condition.^85^, ^86^

#### 2.2.2 Nucleotide excision repair

NER is a highly conserved and versatile pathway that repairs a broad range of DNA helix‐distorting and bulky adducts, including UV‐induced pyrimidine dimers and all platinum‐induced DNA lesions, such as intra‐strand and inter‐strand crosslinks (ICLs).^87^ The repair of a such variety of structurally different substrates involves more than 30 different proteins in a multi‐step fashion. NER proteins are organized in two major pathways: (1) the global genome repair (GGR) effective in the context of a non‐replicating DNA, that slowly controls and repairs the entire genome, preventing mutations and keeping genomic integrity; (2) the transcription coupled repair (TCR) active during DNA transcription, when RNA polymerase is stalled by the DNA damage.^88^ As with other repair pathways, NER relies on damage recognition, excision and release of the 24–32 nucleotides oligomer, subsequent synthesis of the excised sequence and ligation. Briefly, RPA, Xeroderma pigmentosum (XP) group A (XPA), XPC, and transcription factor IIH (TFIIH) cooperate to detect the damage.^89^ TCR‐NER requires Cockayne syndrome group A and Cockayne syndrome group B to detect the lesion, while in GGR‐NER, the XPC and DNA damage‐binding protein B2 (DDB2) participate in the process.^90^ After this different initial recognition step, the two sub‐pathways involve the XPB and XPD helicases with the aim to unwind the DNA double helix around the lesion. The 3′ endonuclease XPG then replaces XPC and recruits the 5′ nuclease ERCC1/XPF a complex, both cleaving the DNA around the lesion and the damaged sequence is removed.^91^ The gap is then filled by POLδ or POLε, with the aid of PCNA and RFC, and the SSB is ligated by DNA ligase III.^92^ Germline mutations in NER genes have been linked to various autosomal recessive disorders in humans, including XP, Cockayne syndrome, and UV‐sensitive syndrome.^93^, ^94^ Patients affected by XP display hyper‐photosensitivity and have a high risk of developing early skin cancer.^95^ Different preclinical and clinical studies highlighted the role of NER in repairing platinum‐induced crosslinks, supporting NER proteins as a potential biomarkers and therapeutic targets.^96^ Cells defective in ERCC1 and XPF genes were 100‐fold more sensitive to cisplatin than the parental line,^97^ as well as cancer cells where the complex ERCC1‐XPF has been silenced.^98^ Data from TCGA reported that approximately 8% of ovarian carcinomas harbor defective mutations in NER genes,^99^ and Ceccaldi and coworkers identified a subgroup of high‐grade serous ovarian cancers characterized by NER alterations and a longer survival (similar to those harboring *BRCA1/BRCA2* mutations) compared to those with wild‐type (WT) NER.^100^ While a defective NER is clearly associated with platinum responsiveness, it is still poorly understood whether and how acquired platinum resistance is correlated with upregulation of NER activity. Induced overexpression of NER genes in colorectal and gastric cancer cells decrease cisplatin response^101^, ^102^ and recently increased NER activity has been reported in ovarian cell line made resistant to cisplatin in vitro.^103^

#### 2.2.3 DNA double‐strand break repair

**FIGURE 3:**
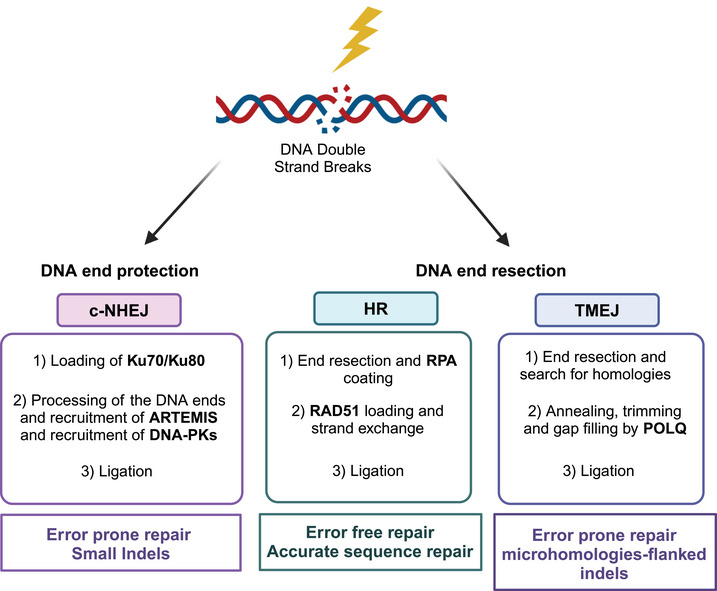
Repair of DNA double strand breaks. This type of damage can be repaired mainly by three pathways that have different initial processing (DNA end resection versus no resection) and are characterized by a different degree of fidelity, as shown in the figure. c‐NHEJ, classical‐non‐homologous‐end‐joining; DNAPKcs, DNA‐protein kinase catalytic subunit; HR, homologous recombination; POL, polymerase; TMEJ, polymerase theta‐mediated end joining. The figure has been created by the authors using BioRender.

DNA‐DSBs are the most deleterious DNA lesions, as they can result in chromosomal aberrations, insertions and deletions and many other mutagenic outcomes.^104^ For these reasons, several mechanisms have been elaborated with different degree of fidelity (Figure 3). Briefly, the three main well‐characterized repair pathways of the DNA‐DSBs are NHEJ, HR, and polymerase theta‐mediated end joining (TMEJ) pathways.^105^

NHEJ ligates the ends of DNA without processing them and is active throughout the different phases of the cell cycle. The first components of the NHEJ recruited at DSB site are the Ku70 and Ku80 subunits, which bind to the broken DNA ends and recruit DNA‐dependent protein kinase catalytic subunit (DNA‐PKcs) leading to the formation of a synaptic complex. This induces translocation of the Ku heterodimers into the DNA duplex, where the broken DNA ends are tethered and ligated. DNA‐PKcs acts as a scaffold protein, promoting the loading of other repair proteins to the site of damage, and it phosphorylates a number of substrates (Ku70, Ku80, Artemis, X‐ray cross‐complementing protein 4 [XRCC4], XRCC4‐like factor, and DNA ligase 4).^106^, ^107^, ^108^, ^109^ This pathway is referred as error prone as not using the homologous template small deletions/insertions at the site of the double strands can occur modifying the original DNA sequence; it is nevertheless responsible for the majority of DNA‐DSBs repair and is quite efficient and most accurate most of the time which explains why cells have evolved in using it.^110^

HR is a conservative error‐free process occurring in the S/G2 phases of the cell cycle, where the sister chromatids are available. It requires resection of the DNA ends, done by the MRN complex (MRE11, RAD50, and NSB1), which generates the 3′‐ssDNA. It also requires BRCA1, BRCA2, and RAD51, a recombinase capable of strand invasion, homology search on the sister chromatid and strand exchange. A detailed description of the HR process is provided in Refs.^111^, ^112^, ^113^ These two repair processes, NHEJ and HR, are responsible for the repair of most DSBs.

TMEJ is defined by its requirement for Polθ. This repair process overlaps the other two processes to a certain extent: the alternative non‐homologous end joining (Alt‐NHEJ) and the microhomology‐mediated end joining pathways.^114^, ^115^ In mammals, TMEJ is essential for viability in cells lacking NHEJ and HR^115^, ^116^ and its inhibition has been suggested as a possible therapeutic opportunity in the settings mentioned.^116^, ^117^, ^118^ HR and TMEJ require 5′–3′ nucleolytic resection of the broken DNA ends to generate ends with 3′‐ssDNA overhangs; these resections generate DNA intermediates that funnel repair to HR and TMEJ, hindering the activation of NHEJ, and have been reported to actually control the DSB repair pathway choice.^119^

NHEJ and HR have been the most widely studied pathways and are thought to be the most important and dominant, while TMEJ was originally believed to act as a backup pathway both for DSB repair when NHEJ and HR were compromised and for the re‐establishment of replication following replication fork collapse.^120^ However, there is now experimental evidence to support its role also in specific settings of DNA metabolism when HR and NHEJ pathways are functional.^121^, ^122^

#### 2.2.4 Fanconi anemia

FA is a rare genetic disorder caused by inactivating mutation of one of the 22 FA genes.^123^ These genes mainly repair the ICLs, even if it has been described its involvement in other genomic integrity maintenance pathways. ICLs are damages that covalently binding both DNA strands and that interfere with DNA replication and genetic transcription impeding the separation of the two DNA strands.^124^ The pathway is active in S phase of the cell cycle, and requires converging replication forks and orchestrating different steps: lesion recognition, DNA incision, lesion bypass, and lesion repair (for a detailed review, please refer to Refs.^123^, ^125^). Briefly, the key event in the process is the mono‐ubiquitination of FANCD2‐I complex. FANCM recruits the FA core complex to the ICL site and this FA core complex ubiquitylates the FANCD2 complex. Ubiquitylated FANCD2 clamps the stalled DNA replication fork and protects it from nucleolytic degradation. The FANCP/SLX4, a DNA endonucleases, will unhook the ICL site by cleaving the surrounding DNA and the resulting a DSB will be repaired by the downstream FA/BRCA proteins using HR and TLS DNA polymerases.^124^, ^125^, ^126^ The FA blocks the activity of alternative low‐fidelity DNA repair pathways (i.e., NHEJ), promoting HR repair.^127^ There is increasing evidence of FA crosstalk and other repair processes, such NER, HR, and translation repair. FA has been shown to maintain genomic stability by ensuring the proper segregation of chromosomes during mitosis.^128^ In addition, ubiquitylated FANCD2 (FANCD2‐Ub) has a crucial role in protecting replication forks from nucleolytic degradation, in conjunction with other repair pathway.^129^

#### 2.2.5 Mismatch repair

MMR detects and fixes the incorrect incorporation of single nucleotides (base‒base mismatch) or mis‐insertion/deletion loops occurring during DNA replication or caused by methylation, oxidation, or inter‐strand crosslinks.^130^, ^131^ In eukaryotic cells, MMR involves different enzymes: MSH2, MSH6, MLH1, and PMS2 that interact forming heterodimers. MSH2 dimerizes with MSH6 or MSH3 forming MutSα and MutSβ, respectively, able to detect mismatches or indel loops. When MutS complex slides along the newly replicated DNA strand and finds the mismatch, it recruits MutL (MutLα, MutLβ, or MutLγ complexes, composed of MLH1 coupled with PMS2 or MLH3)^132^ and creates a nick. Subsequently, DNA is unwound and excised by the exonuclease I (Exo1), that removes short sequence (up to four nucleotides) containing the error, then polymerase δ or polymerase ε with PCNA, RFC, and RPA, correctly resynthesizes the ssDNA sequence and finally ligase I ligates the nick.^69^, ^133^ Mutations in MMR genes can destabilize the genome increasing the mutational rate and inducing microsatellite instability, which favors cancer development.^134^ Inherited mutations in MMR represent the second most common cause of hereditary ovarian cancer, after *BRCA1/BRCA2* mutations, and underlies the Lynch syndrome that predisposes to colorectal, endometrial, and ovarian cancers.^135^ However, MMR alterations can also occur in sporadic tumors, where epigenetic modifications as hypermethylation of hMLH1 promoter, affect protein expression and associated with poorer outcome.^136^, ^137^

#### 2.2.6 Translesion synthesis pathway

DNA lesions persisting in S phase will greatly interfere with DNA replication. The Polδ and Polε polymerases involved in DNA synthesis are high fidelity, but are limited in their ability to accommodate aberrant DNA structures (as in the presence of a DNA damage) and this can lead replication fork stalling, fork collapse, and SSB and DSB generation. To face these problems, cells have evolved the so‐called DNA damage tolerance (DDT) pathways that allow the replication of DNA without repairing the impeding DNA lesions.^138^, ^139^ The TLS is a DDT sub‐pathway that uses low‐fidelity DNA polymerases to insert nucleotides across DNA lesions and/or fill lesion‐containing ssDNA gaps left after replication during G2/M phase.^140^, ^141^, ^142^ The TLS polymerases in human consist of the Y‐family DNA polymerases (Polη, Polι, Polκ, Rev1) and the B‐family polymerase (Polζ) and are considered low fidelity as even if accommodate DNA lesions in their catalytic site, fewer contact with primer/template DNA are formed and are more prone to insert mispairing bases as they lack a 3′‒5′ exonuclease actvity.^143^, ^144^ These properties while enabling replication past sites of DNA damage are associated with an increase rate of mutations.^145^

## 3 DNA DAMAGE RESPONSE IN CANCER AND ITS INHIBITION AS A THERAPEUTIC VALUE

Defects in DDR cause and promote genomic instability. While being an intrinsic property of tumors, it also represents a unique therapeutic opportunity and has become an attractive target for cancer therapy in the last decade. Its inhibition is anticipated to delay the repair of different DNA damages, that is, SSB and DSB, causing persistent unrepair breaks and to interfere with cell cycle progression given the role of its key proteins in the regulation of the cell cycle checkpoints. All these effects sensitize cancer cells to different DNA‐damaging agents, such as RT and chemotherapy, as clearly demonstrated in preclinical and clinical studies, supporting their role in combination therapy.^2^, ^8^, ^37^

The introduction and development of synthetic lethality concept in oncology have allowed the use of drugs in specific tumor genetic context, tailoring anticancer therapy.^146^ From the original observation that poly(ADP‐ribose) polymerase inhibitors were particularly efficacious in *BRCA1/2*‐defective tumors (recently reviewed in Refs.^147^, ^148^), many other synthetic lethal interactions have been looked for and found, in particular within the DDR, considering that is often deregulated, inactivated in tumors. This was also aided by the availability of high‐throughput screening technologies (CRISPR/Cas9 lentivirus screening libraries, FDA‐approved chemical libraries with more than 2500 compounds) that allowed the rapid identification of synthetic lethal interactions.

ATM inhibition has been shown to be in synthetic lethal interaction with PTEN deficiency: PTEN‐lacking prostate cells were more sensitive to ATM inhibitors than PTEN‐proficient cells,^149^ with PARP inhibitors and with MEK1/2 inhibitors. ATM loss‐of‐function is synthetic lethal with MEK1/2 inhibitors^150^ and PARP inhibitors^85^, ^151^ DNA‐PKcs and ATM are in a synthetic interaction as confirmed in a large cell‐based screening.^152^

ATR inhibition has been shown to be lethal in ATM‐ and p53‐deficent cells (i.e., chronic lymphocytic leukemia, pancreatic adenocarcinoma, mantle cell lymphoma, and gastric cancer cells) and the molecular mechanisms underlying seem to depend on the specific cell type.^37^, ^153^, ^154^, ^155^, ^156^ These lethal interactions are displayed when combined with treatment with DNA‐damaging or RS‐stimulating agents, such as IR, camptothecin derivatives, or cisplatin.

Experimental data on the synthetic lethal interaction with Polθ inhibition suggest that HR and NHEJ‐deficient tumors could benefit from Polθ inhibition.^105^, ^116^, ^119^, ^157^, ^158^, ^159^, ^160^, ^161^, ^162^ A CRISPR/Cas9 screening in the cells deficient in Polθ detected 140 genes in which interference impaired cell growth.^163^ Such synthetic lethal interactions are likely to be due to chromosomal abnormalities caused by the loss of TMEJ, which functions as a backup pathway for the repair of DNA‐DSBs in cell deficient in HR and NHEJ pathways. While the synthetic lethality data for Polθ suggest specific settings in which Polθ inhibitors could be beneficial (HR‐deficient tumors, *TP53BP1* mutants or *DNA‐PKcs*‐mutant cancers) and based on the role of Polθ in replication associated lesions, they could also be used in combination therapy with RS inducing agents, such as ATR and topoisomerase inhibitors.^164^ Polθ knockout mice are hypersensitive to IR and bleomycin.^165^ U2OS cells depleted of Polθ through siRNA displayed enhanced sensitivity to camptothecin and etoposide, respectively, a topoisomerase I and topoisomerase II inhibitors.^166^ The authors also reported that breast cancer cells with overexpression of Polθ, and its inactivation sensitizes to topoisomerase inhibitors and the ATR inhibitor VE822.^166^ There is also evidence that deficiency in Polθ can sensitize cells to RT and DNA damaging agents.^167^, ^168^

## 4 TARGETING KEY PLAYERS OF THE DDR

We will here focus on ATM, ATR, Chk1, Wee1, and Polθ inhibitors, reporting their development and the results of the ongoing clinical trials.

### 4.1 ATM inhibitors

Table 1 shows the inhibitors currently in clinical development. All these compounds are ATP competitors, which have been demonstrated to be quite specific against ATM, even if some other PI3K family members could also be affected. All the compounds have reported to sensitize cancer cells to DSB inducers (IR and topoisomerase inhibitors) fostering their clinical development in combination settings. The clinical focus of ATM inhibitors is mainly a combinatorial approach.

**TABLE 1:** Ataxia‐telangiectasia mutated (ATM) inhibitors in clinical development.

Name	NCT number	Phase	Conditions	Combination drug	Study status
M4076 (Lartesertib)	NCT04882917	Phase I	Advanced solid tumors	Monotherapy	Completed
	NCT06433219	Phase II	Ovarian cancer	Tuvusertib, niraparib, lartesertib	Recruiting
	NCT03188965	Phase I	Advanced solid tumor, non‐Hodgkin's lymphoma, mantle cell lymphoma	Tuvusertib, niraparib, lartesertib	Completed
XRD‐0394	NCT05002140	Phase I	Metastasis, locally advanced solid tumor, recurrent cancer	Palliative radiotherapy	Completed
AZD1390	NCT03423628	Phase I	Brain cancer	Radiotherapy	Recruiting
AZD0156	NCT02588105	Phase I	Advanced solid tumors	Olaparib, irinotecan	Completed

*Source*: https://clinicaltrials.gov (August 26, 2024).

M3541 and M4076 represent new class of reversible 1,3‐dihydro‐imidazo[4,5‐c]quinolin‐2‐one compounds; they are potent and selective ATM inhibitors with optimized pharmacological properties with preclinical data supporting their antitumor activity in combination with IR, PARP, and topoisomerase inhibitors.^169^ Both M3541 and M4076 are under clinical development. M3541 (50–300 mg) was administered in combination with fractionated palliative RT (30 Gy in 10 fractions) in 15 patients with solid tumors.^170^ While all the patients reported ≥1 treatment‐emergent adverse event (TEAE), no treatment discontinuation occurred. No grade ≥4 TEAEs were reported and in three patients, complete or partial response were observed. However, no further clinical development of M3541 will be pursued. The results of the part 1A of the phase I study with M4076 were recently reported (NCT04882917).^171^ Twenty‐two patients were treated with M4076 at four different dose levels (100‒400 mg once daily). Dose‐limiting toxicities (DLT) were reported in four patients; six patients had >1 TEAE grade 4, being the most common rash and anemia. The maximum tolerated dose (MTD) found was 300 mg and the steady state plasma concentration reached at 200 mg exceeded the in vivo pChk2 IC50 and preliminary pharmacodynamic data suggest a trend of γH2AX decrease. The multicenter study DDRiver Solid Tumors 320 (NCT05396833) investigated the safety, tolerability, pharmacokinetics, and pharmacodynamics of the ATR inhibitor (tuvusertib) and the ATM inhibitor M4076 in therapy refractory advanced solid tumors.^172^ Five out of the 42 treated patients experienced DLT (three neutropenia grade 3 and 4, two thrombocytopenia grade 2 and 3; frequent TEAEs were anemia, nausea, fatigue, and vomiting).

XRD‐0394, whose chemical structure was recently disclosed, is a potent and specific dual inhibitor of ATM and DNA‐PKcs. This is orally bioavailable small molecule with significantly enhanced tumor cell kill by IR and topoisomerase I in vitro and in vivo^173^; in addition, it showed single‐agent activity and synergy in combination with PARP inhibitors in *BRCA1/2*‐mutated models.^173^ The drug has been evaluated in a phase I trial in combination with palliative RT in metastatic cancer, locally advanced and recurrent cancers. The trial has been completed and results are expected soon.

AZD0156 is a potent and selective bioavailable ATM inhibitor.^174^ It displayed a strong radio‐sensitized effect in vivo and in vitro and it also potentiates the activity of olaparib in a panel of different cancer cell lines and improved its in vivo antitumor activity in triple‐negative breast cancer (TNBC) models.^174^ More recently, its combination with irinotecan provided to be active in different colorectal cancer model.^175^ The AZD0156 safety profile, tolerability, pharmacokinetics, and preliminary efficacy of escalating doses alone or in combination with other drugs have been investigated in a modular phase I trial in patients with advanced malignancies (NCT02588105). The results of module 1 of the trial (AZD0156 in combination with olaparib) has been reported.^176^ Forty‐seven patients were treated in eight cohorts treated with the drug and olaparib. Minor toxicities were nausea and vomiting; the hematological toxicity was the limiting toxicity of the combination. Two confirmed partial responses and one stable disease for 18 months were observed. The drug doses used achieved exposure consistent with its in vitro efficacy and the final results of the study are awaited.

AZD1390 was specifically optimized for blood brain barrier (BBB) penetration as confirmed in cynomolgus monkey brain by positron emission tomography imaging of microdosed ^11^C‐labeled drug.^177^ Based on these data, AZD1390 is in early clinical development as a radiosensitizer in central nervous system malignancies. Recently, using 10 orthotopic glioblastoma (GBM) models, AZD1390 in combination with RT was more effective in *TP53*‐mutant tumors than in *TP53*‐WT patient‐derived xenografts (PDXs). Mechanistic studies suggested that *TP53*‐mutant, but not in *TP53*‐WT, PDXs displayed increased endogenous DNA damage and constitutive ATM signaling.^178^

WSD0628 was shown to potentiate IR in in vivo and in vivo preclinical models of GBM and melanoma.^179^ Its pharmacokinetics profile after oral dosing reveled high level of free drug availability in the brain and in cerebrospinal fluid with little to no Pgp/BCRP substrate liability. A phase 0/I clinical trial of WSD0628 in combination with RT for recurrent brain tumors is ongoing.

### 4.2 ATR inhibitors

The antitumor activity of ATR inhibitors is mainly due on their ability to induce replication fork collapse and DNA‐DSBs accumulation, to inhibit S phase and G2/M checkpoints, to increase RS, and to cause early entry in mitosis and mitotic catastrophe.^180^, ^181^, ^182^ While the *ATR* gene is essential for cell survival, healthy cells are capable of tolerating low protein levels.^183^ Cancer cells rarely display *ATR* mutations and are more susceptible to ATR inhibition since rely on ATR/Chk1 to safely progress through the cell cycle, to tolerate RS and to cope with genomic instability.^184^

The ATR inhibitors currently investigated in clinical trials are summarized in Table 2. Comprehensively, the early phase I/II clinical studies high lightened their good safety profile with manageable hematological and gastric side effects.^185^, ^186^, ^187^, ^188^

**TABLE 2:** Ataxia‐telangiectasia‐and‐Rad3‐related protein (ATR) inhibitors in clinical development.

Name	NCT number	Phase	Conditions	Combination drug	Study status
Art‐0380	NCT04657068	Phase I/II	Advanced or metastatic solid tumors	Gemcitabine, irinotecan	Recruiting
	NCT05798611	Phase II	Solid tumors	Monotherapy	Recruiting
ATRN‐119	NCT04905914	Phase I/II	Advanced solid tumors	Monotherapy	Recruiting
Ceralasertib	NCT05469919	Phase I	Advanced solid malignancies	Monotherapy	Completed
	NCT03527147	Phase I	Non‐Hodgkin's lymphoma; diffuse large B‐cell lymphoma	Acalabrutinib, rituxumab, AZD9150, AZD5153, Hu5F9‐G4	Completed
	NCT03022409	Phase I	Head and neck carcinoma	Olaparib	Completed
	NCT02630199	Phase I	Refractory cancer	Paclitaxel	Completed
	NCT03428607	Phase II	SCLC	Olaparib	Completed
	NCT02937818	Phase II	Platinum refractory small cell lung carcinoma	Olaparib	Completed
	NCT03669601	Phase I	Cancer	Gemcitabine	Recruiting
	NCT04704661	Phase I	Advanced solid tumors expressing HER2	Monotherapy	Recruiting
	NCT03770429	Phase I	Leukemia; myelodysplastic syndrome	Monotherapy	Recruiting
	NCT04550104	Phase I	NSCLC	RT	Recruiting
	NCT02264678	Phase I/ II	Advanced malignancies	Olaparib, AZD1390, AZD5305, durvalumab	Recruiting
	NCT04699838	Phase II	Extensive stage SCLC	Olaparib, carboplatin AZD5305, durvalumab	Recruiting
	NCT03579316	Phase II	Recurrent ovarian, primary peritoneal, or fallopian tube cancer	Adavosertib, olaparib	Recruiting
	NCT04699838	Phase II	SCLC	Cisplatin, carboplatin	Recruiting
	NCT04298008	Phase II	Bile duct cancer; chemotherapy effect	Durvalumab	Recruiting
	NCT04090567	Phase II	Advanced or metastatic germline BRCA‐mutated breast cancer	Olaparib, cedinarib	Recruiting
	NCT03740893	Phase II	Breast neoplasm	Olaparib, durvalumab	Recruiting
	NCT03682289	phase II	Solid tumors	Olaparib, durvalumab	Recruiting
	NCT03801369	phase II	Metastatic triple‐negative breast cancer	Capivasertib	Recruiting
	NCT05582538	phase II	Triple‐negative breast cancer metastatic	Durvalumab, nab‐paclitaxel	Recruiting
	NCT04065269	Phase II	Gynecological cancers	Olaparib, durvalumab	Recruiting
	NCT05941897	Phase II	Advanced or metastatic NSCLC	Durvalumab	Active, not recruiting
	NCT05061134	Phase II	Melanoma	Durvalumab	Active, not recruiting
	NCT04564027	Phase II	Advanced solid tumors	Monotherapy	Active, not recruiting
	NCT04417062	Phase II	Osteosarcoma; osteosarcoma recurrent	Olaparib	Recruiting
	NCT04298021	Phase II	Bile duct cancer	Monotherapy	Active, not recruiting
	NCT03878095	Phase II	Solid neoplasm	Olaparib	Active, not recruiting
	NCT03833440	Phase II	NSCLC	Monotherapy	Active, not recruiting
	NCT03787680	Phase II	Prostate cancer	PARPi	Active, not recruiting
	NCT03462342	Phase II	High‐grade serous carcinoma	Olaparib	Active, not recruiting
	NCT03334617	Phase II	NSCLC	Monotherapy	Active, not recruiting
	NCT03330847	Phase II	Metastatic triple‐negative breast cancer	Olaparib	Active, not recruiting
	NCT05450692	Phase III	Advanced or metastatic NSCLC	Durvalumab	Active, not recruiting
	NCT05514132	Phase I	Advanced solid tumors	Durvalumab	Active, not recruiting
	NCT02223923	Phase I	Solid tumor refractory to conventional treatment	Palliative RT	Active, not recruiting
	NCT03328273	Phase I	Chronic lymphocytic leukemia	Acalabrutinib	Active, not recruiting
Camonsertib (RG6526, RP‐3500)	NCT04855656	Phase I	Advanced solid tumor	RP‐6306, Debio0123	Recruiting
	NCT04972110	Phase I/II	Advanced solid tumor, adult	Niraparib, olaparib	Recruiting
	NCT04497116	Phase I/II	Advanced solid tumor	Talazoparib, gemcitabine	Recruiting
	NCT05566574	Phase I/II	Solid tumor|metastatic cancer	RT	Recruiting
	NCT05405309	Phase I/II	Chronic lymphocytic leukemia	Olaparib	Recruiting
	NCT04589845	Phase II	Solid tumors	Regimen tailored the NGS results	Recruiting
	NCT03337698	Phase I/II	Carcinoma, NSCLC	Multiple immunotherapy–treatment combinations	Active, not recruiting
Gartisertib (M4344)	NCT02278250	Phase I	Solid tumors; advanced solid tumors	Monotherapy	Completed
Elimusertib (BAY1895344)	NCT04095273	Phase I	Advanced solid tumors	Pembrolizumab	Completed
	NCT03188965	Phase I	Advanced solid tumor and lymphoma	Monotherapy	Completed
	NCT04576091	Phase I	Head and neck cancer	Pembrolizumab	Active, not recruiting
	NCT05071209	Phase I	Relapsed or refractory solid tumors	Monotherapy	Active, not recruiting
	NCT04616534	Phase I	Advanced pancreatic and ovarian cancer, and advanced solid tumors	Gemcitabine	Active, not recruiting
	NCT04491942	Phase I	Advanced solid tumors	Monotherapy	Active, not recruiting
	NCT04535401	Phase I	Cancers of the stomach and intestines	Cisplatin, gemcitabine	Active, not recruiting
	NCT04514497	Phase I	Advanced stage solid tumors	Irinotecan, topotecan	Active, not recruiting
Tuvusertib (M1774)	NCT05950464	Phase I	Recurrent ovarian and endometrial cancer	ZEN‐3694 (BET bromodomain inhibitor)	Recruiting
	NCT05396833	Phase I	Metastatic or locally advanced unresectable solid tumors	Lartesertib, avelumab	Recruiting
	NCT05687136	Phase I	Advanced solid tumors	Peposertib	Recruiting
	NCT05882734	Phase I/II	NSCLC	Cemoplimab	Recruiting
	NCT05691491	Phase I/II	Advanced malignant solid neoplasm	Temozolomide	Recruiting
	NCT06433219	Phase II	Ovarian cancer	Niraparib, lartesertib	Recruiting
	NCT05828082	Phase II	Refractory prostate carcinoma	Monotherapy	Recruiting
	NCT05947500	Phase II	Merkel cell skin cancer	Avelumab	Recruiting
	NCT04170153	Phase I	Metastatic or locally advanced unresectable solid tumors	Niraparib	Active, not recruiting

Abbreviations: BRCA, breast cancer; NGS, next generation sequencing; NSCLC, non‐small cell lung cancer; PARPi, poly(ADP‐ribose) polymerase inhibitor; RT, radiotherapy; SCLC, small cell lung cancer.

*Source*: https://clinicaltrials.gov (August 26, 2024).

Berzosertib (M6620, VX‐970, VE‐822) was the first ATR inhibitor evaluated in patients, with the first participant enrolled in a clinical study in 2012 (in NCT02157792 phase I trial). Berzosertib is a potent ATP‐competitive inhibitor of ATR/ATM with over >100‐fold selectivity over ATM, DNA‐PKcs, and other PI3Kα kinases^189^ and strongly reduced the phosphorylation of Chk1, particularly in ATM/p53‐deficient cell lines^190^. In preclinical studies, berzosertib increased the activity of cisplatin, gemcitabine, irinotecan in in vitro and in vivo lung cancer models, and in pediatric solid tumor xenografts.^191^, ^192^, ^193^ The combination with cisplatin resulted promising also in other different tumor types, such as colon cancer and TNBC.^192^, ^194^ Based on these preclinical results, berzosertib has been studied in hundreds of oncological patients in combination with DNA‐damaging chemotherapy (NCT02157792, NCT03896503, NCT03641313, NCT03517969, NCT02627443, NCT02595931, NCT02595892, NCT02567409, and NCT02487095), PARP inhibitors (i.e., veliparib; NCT02723864), radiation (NCT02589522 and NCT04052555), and immunotherapy (i.e., avelumab; NCT04216316). It was well tolerated in monotherapy, as well as in combination with cisplatin or gemcitabine or topotecan.^187^, ^195^, ^196^, ^197^, ^198^, ^199^ Berzosertib in monotherapy was evaluated in a phase I study (NCT02157792) in 17 patients, and one patient with colorectal cancer harboring ATM and ARID1A deficiency reached a complete response.^187^ Combination in the phase I “CHARIOT” study (NCT03641547) assessed the safety, tolerance, and preliminary efficacy of berzosertib in esophageal cancer with RT, and advanced solid tumors with cisplatin and capecitabine.^200^ The combination of berzosertib and gemcitabine improved the median PFS compared to gemcitabine alone in high‐grade platinum‐resistant ovarian cancer.^201^, ^202^, ^203^ The combination of berzosertib with cisplatin and veliparib showed antitumor activity in HR‐deficient patients (NCT02723864),^204^ and patients with recurrent small cell lung cancer (SCLC) showed tumor regression when berzosertib was combined with topotecan (NCT02487095).^199^

Ceralasertib (AZD6738) is a potent, selective, and the first orally available ATR inhibitor, developed by AstraZeneca. At the present, it is under evaluation in numerous trials, including a phase III trial (NCT05450692) in combination with durvalumab (an anti PD‐L1 monoclonal antibody) to evaluate their efficacy compared to docetaxel treatment in patients with advanced or metastatic non‐small cell lung cancer (NSCLC) resistant to immunotherapy and platinum‐based chemotherapy.^205^ Clinical activity of this combination derived from multiple phase II studies in melanoma, gastric cancer, and lung cancer.^206^, ^207^, ^208^ Recent preclinical research showed that intermittent treatment with AZD6738 induces immunomodulatory changes in the tumor microenvironment, suppresses the proliferating CD8+ T cells, and causes an up‐regulation of the cancer inhibitory type I interferon pathway in association with immunotherapy.^209^ Ceralasertib combined with PARPi was found active in *BRCA2*‐mutated TNBC PDX^210^ and in *BRCA1/2* mutant high grade serous ovarian cancer (HGSOC) PDX models progressing on PARPi with a significant tumor regression and increase in overall survival (OS).^211^ These latest results fostered the phase II CAPRI trial (NCT03462342), where the combination of ceralasertib plus olaparib is being evaluated in recurrent HGSOC patients in three cohorts: (1) platinum‐sensitive, (2) platinum‐resistant (both 1 and 2 are independent of HR status), and (3) platinum‐sensitive disease that has progressed after PARPi treatment in HR‐deficient patients. The results of the second cohort (i.e., genetically unselected, recurrent platinum resistant, *n* = 12)^212^ reported stable disease in 9 out of 12 subjects, while three progressed under treatment. Overall median Progression Free Survival (PFS) was 4.2 months, while the patients with germline or somatic *BRCA1* mutations (*n* = 3) had PFS events at 8.2, 9.8, and 3.6 months, respectively. The combination was well tolerated with a safety profile similar to that of olaparib single agent. In a more recent analysis of a phase II biomarker‐driven umbrella study (NCT03428607), in platinum‐resistant SCLC patients, the combination of olaparib plus ceralasertib showed limited efficacy in patients without DDR alteration compared with olaparib monotherapy in subjects with DNA repair alterations.^213^ The importance of selecting patients with specific genomic alterations is highlighted by the results from the third cohort of the CAPRI study showing that 50% of evaluable patients (*n* = 6) experienced a partial response, despite the fact that all patients were progressing on a PARPi, suggesting the addition of ceralasertib to olaparib, re‐sensitized PARPi‐resistant tumors to olaparib.^214^

Camonsertib (RG6526, RP‐3500) is a potent ATR inhibitor with a demonstrated dose‐dependent ability to reduce Chk1 phosphorylation and increase γH2AX levels, two markers of ATR inhibitor activity.^215^ It was developed using a synthetic lethality approach based on CRISPR/Cas9 for the treatment of tumors with specific genomic alterations in DDR genes.^216^ It is in phase I and II trials for treatment of advanced solid tumors as monotherapy, in combination with PARPi (NCT04972110) or gemcitabine (NCT04497116).

A recent preclinical study reported that the combination of camonsertib with lunresertib (RP‐6306), a PKMYT1 inhibitor synergistically increased cytotoxicity in CCNE1 amplified than in WT ovarian and endometrial cancer models.^217^ Camonsertib monotherapy induced significant tumor growth inhibition in an ATM‐deficient colorectal in vivo model.^215^ Preliminary results demonstrate the safety, tolerability, and early efficacy of the camonsertib/lunresertib combination across multiple tumor types and genotypes, with the strongest antitumor activity in gynecologic tumors.^218^ Data from the from module 1 of TRESR phase I/II trial (NCT04497116) involving 120 patients with advanced solid tumors with DDR alterations, showed that camonsertib was well tolerated; anemia was the most common side effect observed (32% grade 3). Overall clinical response, clinical benefit, and molecular response rates were, respectively, 13%, 43%, and 43% with particular benefit observed in ovarian.^219^

Elimusertib (BAY‐1895344), developed by Bayer Pharmaceuticals, demonstrated a strong antitumor activity especially in preclinical models with DDR defects.^220^, ^221^ Elimusertib monotherapy was very active in pediatric solid cancer PDXs; in addition, it was active in resistant models, suggesting elimusertib could by‐pass drug resistance.^222^ Synergistic activity was observed when elimusertib was combined in in vitro and in vivo models with RT, cisplatin, anti‐androgens, olaparib, and immune checkpoint inhibitors.^221^ The first in‐human trial of elimusertib (NCT03188965) reported an overall good tolerability, with manageable and reversible hematologic toxicities as most common side effects. Antitumor activity against advanced solid tumors and lymphomas with DDR defects, including ATM loss of function alterations, was observed. Four out of 20 subjects achieved partial response, while eight achieved stable disease with a median duration of response of 11.25 months, and 3.6% showed durable objective responses, exceeding 3 years across a subset of molecularly selected cancer types.^187^ Preliminary data from PEPN2112 (NCT05071209) trial, an ongoing phase I study of elimusertib monotherapy in patients with different types of relapsed or refractory tumors, demonstrated primarily hematologic toxicities.^223^ An ongoing phase I trial (NCT04576091) is evaluating stereotactic body RT combined with elimusertib and pembrolizumab for patients with recurrent head and neck squamous cell carcinoma.^224^

Gartisertib (M4344) was developed by Vertex Pharmaceuticals. In several cancer cell lines, it was reported that high RS condition and neuroendocrine gene expression signature were associated with a better response to gartisertib.^225^ In addition, it was highly synergistic with a broad range of common clinical DNA‐damaging agents and induced RS in several cancer cell lines, patient‐derived prostate tumor organoids, and primary SCLC cell line‐mouse xenografts.^225^ Gartisertib monotherapy has been evaluated in a phase I clinical trial now completed (NCT04095273) aimed at evaluating the safety, pharmacokinetics, pharmacodynamics, and antitumor activity in combination with carboplatin in 97 patients with advanced solid tumors. Gartisertib was well tolerated, but it induced an unexpected, transient, liver toxicity. Partial response observed was observed in 6.3% of the cases, three patients experienced stable disease (3.1%). However, prolonged partial response and stable disease did not appear to be associated with biomarker status.^226^

Tuvusertib (M1774) is a small molecule, ATR inhibitor, developed by EMD Serono, active at nM concentrations.^227^ Preclinical studies support its combination with topotecan, irinotecan, etoposide, cisplatin, lurbinectedin, and talazoparib. Tuvusertib was shown to reverse chemoresistance to DNA‐damaging agents in cancer cells lacking SLFN11.^227^ In the phase I study DDRiver Solid Tumors 301 (NCT04170153) for advanced solid tumors, tuvusertib will be evaluated alone or in combination with niraparib in patients with DDR alterations. As regards clinical efficacy one patient with platinum‐ and olaparib‐resistant *BRCA* WT ovarian cancer achieved a partial response and tumors with ARID1A, ATRX, and DAXX mutations seemed to be more sensitive to the drug.^228^ Several phase I/II studies are ongoing to evaluate the safety and tolerability of tuvusertib in combination with immune modulators.

ART0380 is an ARTIOS compound, whose preclinical and preliminary pharmacokinetics and pharmacodynamics clinical data suggest to be rapidly absorbed, leading to a high concentration able to promote apoptosis in DDR defective tumors; it is rapidly eliminated, potentially preserving from toxicity. ART0380 combined with gemcitabine, show encouraging results in preclinical models. A phase I/II study (NCT04657068) in combination with gemcitabine in advanced/metastatic platinum‐resistant ovarian cancer is recruiting patients.^229^ In the dose‐escalation module, the combination with gemcitabine showed a good safety profile. The hematological toxicities reported were manageable and reversible. ART0380 also demonstrated good pharmacodynamic and PK profiles.^230^ Preliminary data from this trial also report synergistic effect in combination with gemcitabine or irinotecan, olaparib, or anti‐PD1.^229^

ATRN‐119 is a small molecule studied by Atrin Pharmaceuticals, and the first macrocyclic ATRinhibitor to enter clinical trials. Currently, it is involved in a phase I/II study (NCT04905914) recruiting subjects with advanced solid tumors to investigate the safety profile, pharmacokinetic properties, preliminary antitumor efficacy, and biomarker profile. In the early 12‐patient cohort, ATRN‐119 once daily administered was well tolerated. Two patients achieved stable disease, one patient received the dose 50 mg and progressed after 112 days, and one patient at the dose level 200 mg remained on treatment up to 118 days.^231^

### 4.3 Chk1 inhibitors

Chk1 and Wee1 inhibitor treatments in cells have been shown to increase origin firing, to inhibit both S and G2 checkpoints causing a RS and premature mitosis entry, leading to DNA‐DSB induction and to mitotic catastrophe.^63^ The development of these inhibitors has mainly focus on monotherapy in tumors with specific DNA genetic backgrounds, in combination with other DNA‐damaging agents and recently in a PARPi‐resistant setting. Indeed, preclinical evidence suggest that many of the mechanisms of PARPi could be counteracted by inhibition of ATR‒Chk1‒Wee1 axis (for review, see Refs.^63^, ^232^).

The chemical and preclinical data on Chk1 inhibitors has been recently reviewed.^233^ Table 3 summaries the Chk1 in clinical development.

**TABLE 3:** Chk1 inhibitors in clinical development.

Name	NCT number	Phase	Conditions	Combination drug	Study status
GDC‐0425	NCT01359696	Phase I	Refractory solid tumors or lymphoma	Monotherapy, gemcitabine	Completed
GDC‐0575	NCT01564251	Phase I	Lymphoma, solid tumor	Monotherapy, gemcitabine	Completed
PEP07	NCT05659732	Phase I	Advanced cancer	Monotherapy	Recruiting
	NCT05983523	Phase I	Advanced or metastatic solid tumors	Monotherapy	Not yet recruiting
MK‐8776 (SCH 900776)	NCT00779584	Phase I	Hodgkin disease, lymphoma, non‐Hodgkin	Monotherapy, gemcitabine	Completed
	NCT01870596	Phase II	Acute myeloid leukemia	Monotherapy, cytarabine	Completed
	NCT00907517	Phase I	Acute leukemias	Monotherapy, cytarabine	Terminated
LY2603618	NCT00415636	Phase I	Cancer	Pemetrex	Completed
	NCT01296568	Phase I	Advanced cancer	Pemetrex, gemcitabine	Completed
	NCT01358968	Phase I	Cancer	Desipramine, pemetrexed	Completed
	NCT01341457	Phase I	Solid tumors	Gemcitabine	Completed
	NCT00839332	Phase I/II	Pancreatic cancer	Gemcitabine	Completed
	NCT01139775	Phase I/II	Non‐small cell lung cancer	Pemetrex, cisplatin	Completed
	NCT00988858	Phase I	Non‐small cell lung cancer	Pemetrex	Completed
LY2880070	NCT02632448	Phase I/II	Advanced or metastatic cancer	Monotherapy, gemcitabine	Recruiting
	NCT05275426	Phase II	Ewing sarcoma|Ewing‐like sarcoma	Monotherapy, gemcitabine	Recruiting
PREXASERTIB (LY2606368)	NCT02873975	Phase II	Advanced cancers	Monotherapy	Completed
	NCT02778126	Phase I	Advanced cancer	Monotherapy	Completed
	NCT02649764	Phase I	Relapsed or refractory acute myeloid leukemia, high‐risk myelodysplastic syndrome	Cytarabine, fludarabine	Completed
	NCT02860780	Phase I	Advanced cancer and metastatic cancer	Ralimetinib	Completed
	NCT03057145	Phase I	Solid tumor	Olaparib	Completed
	NCT02808650	Phase I	Recurrent or refractory pediatric tumors	Monotherapy	Completed
	NCT02514603	Phase I	Advanced tumors	Monotherapy	Completed
	NCT02555644	Phase I	Head and neck neoplasms	Cisplatin, cetuxumab	Completed
	NCT02124148	Phase I	Advanced solid tumors	Cisplatin, cetuxumab	Completed
	NCT01115790	Phase I	Advanced cancer	Monotherapy	Completed
	NCT03414047	Phase II	Ovarian cancer	Monotherapy	Completed
	NCT02735980	Phase II	Small cell lung cancer	Monotherapy	Completed
	NCT04032080	Phase II	Triple‐negative breast cancer	LY3023414	Completed
	NCT05548296	Phase I/II	Platinum‐resistant ovarian carcinoma, endometrial and urothelial carcinoma	Gemcitabine	Recruiting
	NCT04023669	Phase I	Refractory or recurrent group 3/group 4 or SHH medulloblastoma brain	Cyclophosphamide, gemcitabine	Active, not recruiting
	NCT04095221	Phase I/II	Desmoplastic small round cell tumor, rhabdomyosarcoma	Irinotecan, temozolomide	Active, not recruiting
	NCT02203513	Phase II	BRCA1/2‐mutated cancer	Monotherapy	Terminated
	NCT03735446	Phase I	Acute myeloid leukemia|myelodysplastic syndromes	Mitoxantrone, etoposide, cytarabine	Terminated
BBI‐355	NCT05827614	Phase I/II	Locally advanced or metastatic non‐resectable solid tumors harboring oncogene amplifications	Erlotinib, futibatinib	Recruiting
PF‐00477736	NCT00437203	Phase I	Advanced solid tumors	Gemcitabine	Terminated
SRA737	NCT02797964	Phase I/II	Advanced solid tumors or non‐Hodgkin's lymphoma	Monotherapy	Completed
	NCT02797977	Phase I/II		Gemcitabine	Completed
AZD7762	NCT00413686	Phase I	Solid tumors	Monotherapy, gemcitabine	Completed
	NCT00937664	Phase I	Advanced solid malignancies	Monotherapy, gemcitabine	Terminated
	NCT00473616	Phase I	Advanced solid malignancies	Monotherapy, irinotecan	Terminated

Abbreviation: SHH, sonic hedgehog.

*Source*: https://clinicaltrials.gov (August 26, 2024).

GCD‐0425 is an orally bioavailable, highly selective small inhibitor of Chk1. In preclinical studies, it was shown to potentiate the activity of gemcitabine in in vitro and in vivo models, with the induction of mitotic catastrophe and increased in DNA damage.^234^ These data prompted its evaluation in a phase I trials in combination with gemcitabine. Forty patients were treated with this combination and an increase in bone marrow toxicities were reported as compared to the expected with gemcitabine alone. Neutropenia and thrombocytopenia were manageable, but were grade 3 or 4 in 40% and 15% of patients, respectively. Hints of clinical activity were observed with 20% of patients remaining on study for more than 6 months and three PRs were reported.^235^

GCD‐0575 is a very selective oral small‐molecule Chk1 inhibitor showing tumor shrinkage and growth delay in several xenograft models. Its safety, tolerability, and pharmacokinetic properties alone and in combination with gemcitabine have been evaluated in a phase I trial.^236^ While it could be safely administered as monotherapy, its combination with gemcitabine resulted in poor tolerability and in limited clinical activity.^236^

PEP07 is an orally available brain‐penetrant selective Chk1 inhibitor that is entering first in human clinical studies in several advanced tumors.

MK‐8776 is highly selective for Chk1 compared to Chk2 and CDK. Preclinical studies demonstrated its ability to enhance the cytotoxicity of hydroxyurea, gemcitabine and IR in vitro and in vivo without increase in normal tissue toxicity.^237^ A phase I (NCT00907517) study conducted in 24 patients with relapsed and refractory acute leukemias treated with cytarabine and escalating doses of MK‐8776 showed Dose Limiting Toxicity (DLT) consisting of QT interval prolongation and grade 3 palmar‐plantar erythrodysesthesia at the flat dose (140 mg) of MK‐8776. Molecular analyses showed increased phosphorylation of H2AX after drug administration beginning at 40 mg/m^2^, consistent with unrepaired DNA damage. Eight of 24 (33%) patients treated with 40 mg/m^2^ or higher doses reached complete remissions fostering the development of a phase II trial of cytarabine ± MK‐8776 at a recommended flat dose of 100 mg.^238^ In another phase I study conducted in 43 patients with advanced solid tumors or lymphoma, MK‐8776 was administered as monotherapy or in combination with gemcitabine 800 mg/m^2^ (NCT00779584). The treatment was tolerated with some toxicities as QTc prolongation (19%), nausea (16%), fatigue (14%), and constipation (14%) as monotherapy and fatigue (63%), nausea (44%), decreased appetite (37%), thrombocytopenia (32%), and neutropenia (24%) and transient QTc prolongation (17%) when combined with gemcitabine. Again, biological activity was evaluated as phosphorylation of H2AX. Of 30 patients evaluable for response, two showed partial response, and 13 exhibited stable disease.^239^ NCT01870596 is a phase II trial in which patients with relapsed or primary refractory AML randomized to receive either cytosine arabinoside with MK‐8776 or cytosine arabinoside alone. Response rates and survival were similar in the two groups in spite the evidence that Chk1 inhibition augmented DNA damage in circulating leukemic blasts.^240^

Rabusertib (LY2603618) was developed by Lilly Research Laboratories as a clinical candidate with Chk1‐inhibitory potency and a reduced risk of cardiac toxicity. LY2603618 underwent seven phase I/II trials in patients with solid cancers. Most of them were testing the combination of LY2603618 with cytotoxic agents (pemetrexed and gemcitabine); however, the efficacy of the combinations was not improved, while increased toxicity was reported (thromboembolism in combination with pemetrexed and cisplatin).^241^, ^242^, ^243^, ^244^, ^245^, ^246^ The drug has been discontinued.

Prexasertib (LY2603618) is a dual Chk1/2 ATP‐competitive inhibitor induced mitotic catastrophe and apoptosis in cancer cells and showed synergistic effect in combination with both cisplatin and PARPi in in vivo models.^247^, ^248^ Currently, LY2606368 is the most clinically advanced Chk1 inhibitor, with a total of 18 clinical trials (Table 3). Prexasertib could be combined with cisplatin, cetuximab, and 5‐fluorouracil, even if its schedule was a key determinant of the tolerability and feasibility of the combinations (NCT02124148). Hematologic toxicity was the most frequent adverse events (AEs); it was dose limiting and reversible.^249^ Prexasertib could be safely combined with attenuated doses of olaparib. In *BRCA*‐mutant ovarian cancers who have previously progressed on a PARPi, the combination showed hints of antitumor activity; in addition, pharmacodynamic studies on tumor biopsies showed target engagement with RAD51 foci formation and increased expression of γH2AX, pKAP1, and pRPA after combined treatment.^250^ In a phase II study conducted on 169 patients with platinum‐resistant/refractory ovarian cancer (NCT03414047), prexasertib demonstrated durable single agent activity regardless of clinical characteristics, *BRCA* status, or prior therapies, including PARPi. No correlation with genomic alterations in responders versus non‐responders was found.^251^ In another phase II trials conducted on patients with breast or ovarian cancer (NCT02203513), transcriptomic analysis revealed high levels of DNA replication‐related genes (i.e., POLA1, POLE, GINS3) associated with lack of clinical benefit, suggesting that POLA1 expression may predict Chk1 inhibitors resistance, and that its inhibition may improve the efficacy of prexasertib monotherapy.^252^ Prexasertib is now in clinical investigation as monotherapy in advanced solid tumors with genetic alterations in the HR pathway, RS, or with *CCNE1* amplification (NCT02873975).

LY2880070 is a selective ATP‐competitive Chk1 inhibitor. Although no preclinical data are available—at the best of our knowledge—it is now under clinical investigation in different solid tumor. In a phase I study, it was combined with gemcitabine in metastatic PDAC patients (NCT02632448); however, due to drug‐related grade 3 AE, the trial was discontinued with no evidence of clinical activity in patients treated with the combination.^253^

SRA737 is orally bioavailable Chk1 inhibitor shown to be active as monotherapy in *CCNE1* amplified models, to be synergic with PARPi and to be active in PARPi‐resistant *BRCA*‐mutant PDX models.^254^ Phase I/II trial (NCT02797964) was well tolerated at doses that reached relevant dose concentration; however, no clinical activity was observed.^255^ Hints of activity were observed when combined with low‐dose gemcitabine in anogenital and other solid tumors.^256^

### 4.4 Wee1 inhibitors

The chemical and biological characteristics of Wee1 inhibitors have been recently published.^257^ These inhibitors have been shown to have promising antitumor activity, but an increase in adverse effects (myelosuppression) from monotherapy to schedules with chemotherapy has been reported in both preclinical and clinical studies. These results have led to the search for potential biomarker of Wee1 response to better stratify patients. Among the biomarkers proposed there are *CCNE1* amplification, *BRCA* mutations and *TP53* mutation; however, biomarker‐driven studies are quite limited.^258^ The Wee1 inhibitors in clinical development are shown in Table 4.

**TABLE 4:** Wee1 inhibitors in clinical development.

Name	NCT number	Phase	Conditions	Combination drug	Study status
Adavosertib AZD1775	NCT01748825	Phase I	Solid tumors	Monotherapy	Completed
	NCT03313557	Phase I	Solid tumors	Monotherapy	Completed
	NCT02511795	Phase I	Refractory solid tumors	Olaparib	Completed
	NCT02482311	Phase I	Advanced solid tumors	Monotherapy	Completed
	NCT03333824	Phase I	Advanced solid tumors	Monotherapy	Completed
	NCT02610075	Phase I	Advanced solid tumors	Monotherapy	Completed
	NCT02448329	Phase II	Advanced gastric cancer	Paclitaxel	Completed
	NCT02207010	Phase 0	Glioblastoma	Monotherapy	Completed
	NCT02593019	Phase II	Relapsed small cell lung cancer patients	Monotherapy	Completed
	NCT03012477	Phase II	Triple‐negative metastatic breast cancer	Cisplatin, RT	Completed
	NCT02341456	Phase II	Advanced solid tumors	Carboplatin, paclitaxel	Completed
	NCT03253679	Phase II	Advanced solid neoplasm	Monotherapy	Completed
	NCT02906059	Phase I	Metastatic colorectal cancer	Irinotecan	Completed
	NCT02194829	Phase I/II	Metastatic pancreatic adenocarcinoma	Gemcitabine	Completed
	NCT03028766	Phase I	Head and neck tumors	Cisplatin, RT	Completed
	NCT02513563	Phase II	Lung cancer	Carboplatin, paclitaxel	Completed
	NCT02666950	Phase II	Advanced acute myeloid leukemia or myelodysplastic syndrome	Monotherapy, cytarabine	Completed
	NCT02037230	Phase I/II	Pancreatic cancer	Monotherapy, gemcitabine, RT	Completed
	NCT01164995	Phase II	Epithelial ovarian cancer	Carboplatin	Completed
	NCT03579316	Phase II	Ovarian, primary peritoneal, or fallopian tube cancer	Olaparib	Completed
	NCT04462952	Phase I	Advanced solid tumors	Monotherapy	Completed
	NCT02508246	Phase I	Head and neck tumors	Docetaxel, cisplatin	Completed
	NCT02272790	Phase II	Ovarian, fallopian tube, peritoneal cancer	Carboplatin, paclitaxel, gemcitabine, or PDL	Completed
	NCT01922076	Phase I	Astrocytoma, glioma, oligoastrocytoma	RT	Completed
	NCT02095132	Phase I	Relapsed or refractory solid tumors	Irinotecan	Completed
	NCT02937818	Phase II	Platinum refractory small cell lung cancer	Durvalumab, tremelimumab, olaparib	Completed
	NCT04590248	Phase II	Uterine serous carcinoma	Monotherapy	Completed
	NCT01827384	Phase II	Advanced malignant solid neoplasm	Carboplatin	Completed
	NCT00648648	Phase II	Advanced solid tumors	Monotherapy, gemcitabine, cisplatin, carboplatin	Completed
	NCT01357161	Phase II	Ovarian cancer	Carboplatin, paclitaxel	Completed
	NCT02813135	Phase I	Pediatric cancer	Monotherapy	Recruiting
	NCT02465060	Phase II	Refractory solid tumors, lymphomas, or multiple myeloma	Monotherapy	Recruiting
	NCT03668340	Phase II	Uterine cancer	Monotherapy	Active, not recruiting
	NCT03330847	Phase II	Metastatic triple‐negative breast cancer	Olaparib	Active, not recruiting
	NCT04439227	Phase II	Advanced malignant solid neoplasm and lymphoma	Monotherapy	Active, not recruiting
	NCT02617277	Phase I	Advanced solid tumors	Durvalumab, tremelimumab, olaparib	Active, not recruiting
	NCT04197713	Phase I	Advanced solid tumors	Olaparib	Active, not recruiting
	NCT02101775	Phase II	Ovarian, fallopian tube, peritoneal cancer	Gemcitabine	Active, not recruiting
	NCT02546661	Phase I	Invasive bladder cancer	MEDI4736	Active, not recruiting
	NCT02585973	Phase I	Carcinoma, squamous cell of head and neck	Cisplatin, RT	Active, not recruiting
	NCT02659241	Early phase I	Ovarian, fallopian tube, peritoneal cancer	Phase II	Active, not recruiting
	NCT01849146	Phase I	Newly diagnosed or recurrent glioblastoma	RT, temozolomide	Active, not recruiting
	NCT03284385	Phase II	Advanced solid tumors with SETD2 mutation	Monotherapy	Active, not recruiting
	NCT04460937	Phase I	Esophageal and gastroesophageal cancers	RT	Active, not recruiting
	NCT03718143	Phase II	Acute myeloid leukemia, myelodysplastic syndrome and myelofibrosis	Monotherapy	Terminated
	NCT02688907	Phase II	Relapsed small cell lung cancer patients	Monotherapy	Terminated
	NCT02087241	Phase II	Stage IV non‐squamous non‐small cell lung cancer	Monotherapy, pemetrex	Terminated
	NCT02087176	Phase II	Previously treated non‐small cell lung cancer	Docetaxel	Terminated
	NCT03345784	Phase I	Malignant female reproductive neoplasms	Cisplatin, RT	Terminated
	NCT02576444	Phase II	Cancer	Olaparib	Terminated
	NCT02381548	Phase I	Leukemia	Belinostat	Terminated
	NCT04949425	Phase I	Advanced solid tumors	Monotherapy	Terminated
	NCT05008913	Phase I	Advanced solid tumors	Monotherapy	Terminated
	NCT02196168	Phase II	Recurrent or metastatic head and neck cancer	Cisplatin	Terminated
	NCT01076400	Phase I/II	Cervical cancer	Topotecan, cisplatin	Terminated
	NCT01047007	Phase I	Solid tumors	5‐FU, cisplatin	Terminated
	NCT02791919	Phase I	Relapsed or refractory acute myeloid leukemia	Fludarabine, cytarabine, filgrastim	Withdrawn
	NCT05212025	Phase II	Pancreatic cancer		Withdrawn
Azenosertib ZN‐C3	NCT04158336	Phase I	Solid tumor	Monotherapy	Recruiting
	NCT04516447	Phase I	Ovarian cancer	Chemotherapy, bevacizumab	Recruiting
	NCT05682170	Phase I/II	Acute myeloid leukemia	BCL‐2 inhibitor ZN‐d5	Recruiting
	NCT05743036	Phase I/II	Metastatic colorectal cancer	Encorafenib/cetuximab	Recruiting
	NCT06015659	Phase II	Pancreatic cancer|	Gemcitabine	Recruiting
	NCT06351332	Phase I/II	Breast triple breast cancer	Carboplatin, pembrolizumab	Recruiting
	NCT04814108	Phase II	Uterine serous carcinoma	Monotherapy	Active, not recruiting
	NCT04833582	Phase I/II	Osteosarcoma	Gemcitabine	Active, not recruiting
	NCT05128825	Phase II	Ovarian, fallopian tube or primary peritoneal cancer	Monotherapy	Active, not recruiting
	NCT05198804	Phase I/II	Platinum‐resistant ovarian cancer	Monotherapy, niraparib	Active, not recruiting
	NCT06364410	Phase I	Solid tumors	Trastuzumab, deruxtecan	Not yet recruiting
	NCT06369155	Phase II	Uterine cancer	Monotherapy	Not yet recruiting
	NCT04972422	Phase I	Solid tumors	Monotherapy	Unknown
	NCT05368506	Early phase I	Metastatic triple‐negative breast cancer and ovarian cancer	Monotherapy	Withdrawn
	NCT05431582	Phase I	CCNE1 amplified and TP53 mutant solid tumors	Monotherapy, bevacizumab, pembrolizumab	Withdrawn
Debio 0123	NCT03968653	Phase I	Advanced solid tumors	Carboplatin	Recruiting
	NCT05109975	Phase I	Advanced solid tumors	Monotherapy	Recruiting
	NCT05765812	Phase I/II	Glioblastoma, astrocytoma	RT, temozolomide	Recruiting
	NCT05815160	Phase I	Small cell lung cancer progressing after platinum	Carboplatin, etoposide	Recruiting
	NCT04855656	Phase I	Advanced solid tumors	RP‐6306, RP‐3500	Recruiting
IMP768	NCT04768868	Phase II	Advanced solid tumors	Monotherapy	Recruiting
SC0191	NCT06363552	Phase I	Metastatic colon cancer	Monotherapy, bevacizumab, 5‐FU/LV	Not yet recruiting
	NCT06055348	Phase I/II	Ovarian cancer	Gemcitabine, paclitaxel	Not yet recruiting
SY‐4835	NCT05291182	Phase I	Advanced solid tumor	Monotherapy	Recruiting

Abbreviations: 5FU/LV, 5‐fluorouracil/folinic acid; PDL, pegylated liposomal doxorubicin; RT, radiotherapy.

*Source*: https://clinicaltrials.gov (August 26, 2024).

Adavosertib (AZD1775, MK1775) is a potent, selective ATP‐competitive Wee1 inhibitor developed by AstraZeneca that has been shown to potentiate the activity of various DNA‐damaging chemotherapeutic agents in vitro and in vivo.^259^, ^260^, ^261^ In a phase I study conducted in recurrent GBM (NCT02207010), 20 patients received a single dose of AZD1775 prior to tumor resection and were enrolled in either a dose‐escalation arm or a time‐escalation arm. This study provided the first evidence of clinical biological activity in human GBM, where the inhibition of the Wee1 pathway resulted in abrogation of G2 arrest, increase DSBs breakage, and programmed cell death with no drug‐related AEs associated.^262^ A phase I trial conducted in 25 patients with refractory solid tumors (NCT01748825), enrolled six patients with *BRCA*‐mutant solid tumors at the maximum tolerated dose and two patients were confirmed to have a partial response, one with head and neck cancer and one with ovarian cancer. The most common toxicities were myelosuppression (including anemia, neutropenia, and thrombocytopenia) and diarrhea. The DLT were supraventricular tachyarrhythmia (*n* = 1) and myelosuppression (*n* = 1). Biological activity was demonstrated by reduced levels of pY15‐Cdk and increased levels of γH2AX.^263^ Encouraging results after adavosertib monotherapy have been obtained also in a phase II study conducted in patients with uterine serous carcinoma (NCT03668340).^264^ Ten of the 34 evaluable patients responded to the treatment, while 16 were progression free at 6 months. The main AEs included diarrhea (76.5%), fatigue (64.7%), nausea (61.8%), and hematologic AEs. In this study, no correlation of clinical activity with specific molecular alterations were observed.^264^

A dose‐escalation study of adavosertib in combination with radiation was conducted in patients with locally advanced pancreatic cancer (NCT02037230). The combination was well tolerated with neutropenic sepsis/thrombocytopenia (*n* = 1), abnormal liver function test (*n* = 1), anorexia/nausea (*n* = 3), fatigue (*n* = 2), abdominal pain (*n* = 1), and mental state disturbance (*n* = 1) as toxic effect, and the OS was higher than prior results combining gemcitabine with radiation therapy. Wee1 inhibition was demonstrated by decreased phosphorylation of CDK1.^265^

A phase II study conducted in *p53*‐mutated platinum refractory or resistant ovarian cancer AZD1775 enhanced carboplatin efficacy and demonstrated manageable toxicity; fatigue (87%), nausea (78%), thrombocytopenia (70%), diarrhea (70%), and vomiting (48%) were the most common AEs.^266^ As a whole, the clinical study support adavosertib activity in advanced solid tumors, with best response observed in gynecologic cancers.^258^ Combination with different cytotoxic, targeted agents and immune‐suppressive agents are ongoing, but increased toxicity has been reported with the need to optimize treatment schedules.^258^ The drug has been discontinued.

Azenosertib (ZN‐c3) is a more selective ATP‐competitive Wee1 inhibitor developed by Zentalis, currently under clinical investigation both as monotherapy or in combination with anticancer drugs.^267^ All trials are ongoing, but preliminary showed that azenosertib is more active in *CCNE1* overexpressing ovarian cancer cells, suggesting that a better stratification of patients could potentially disclose drug activity.^268^

Recently new, more specific Wee1 inhibitors with a better toxicological profile have been developed: Debio 0123,^269^ IMP7068,^270^, ^271^ SC0191,^272^ and SY‐4835. These drugs are on clinical testing in solid tumors and the preliminary data are encouraging.

In a CRISPR/Cas9 screening, a synthetic lethal interaction between the Wee1‐like kinase PKMYT1 and *CCNE* overexpression was found.^273^ In the same manuscript, the PKMYT1 inhibitor (RP‐360) was found very active in in vivo xenografts and PDX models alone and in combination with gemcitabine. These data foster its clinical development and now there are six phase I and II trials testing the drug alone or in combination with chemotherapy (NCT05147350, NCT04855656, NCT05147272, NCT06107868, NCT05605509, and NCT05601440).

### 4.5 Polθ inhibitors

Polθ is a multifunctional protein, with two distinct activities amenable to inhibition: the Polθ PolD polymerase activity, required for TMEJ in biochemical and cellular assays, and the Polθ HDL helicase and ATPase activities, responsible for the removal of RPA bound to resected DNA ends, for the identification of micro‐homologies and for blocking non‐productive intramolecular primed synthesis. The Polθ PolD domain contains two well‐characterized ligand binding sites (the active site and the allosteric site). While the majority of inhibitors of DNA polymerase interfere with the enzymatic activity by binding the active site of the protein, Polθ PolD is very plastic and the structure of the active residues in the pocket can modify the intramolecular interactions among the incoming nucleotide and the Polθ residues making it harder to design a specific inhibitor. The allosteric site has been recently disclosed by co‐crystallization experiments conducted with the hit compounds deriving from a high throughput screening approach on Polθ‐PolD (ART558 and RP6685).^274^, ^275^, ^276^ Studies on the interaction of the inhibitors with the allosteric site have defined a 3D pharmacophore model available for ligand‐based studies ^275^ Details of chemical structures of the different Polθ inhibitors available has been recently published.^275^ There are few clinical settings in which inhibition of Polθ is likely to be effective and much more data are necessary to define the best ones.

We will briefly discuss their chemical, biochemical, biological activities, and their clinical development (Table 5).

**TABLE 5:** Polθ inhibitors in clinical development.

	NCT number	Phase	Conditions	Combination drug	Study status
Novobicin	NCT05687110	Phase I	Metastatic and unresectable solid neoplasm with DNA damage response alteration	Monotherapy	Suspended
ART6043	NCT05898399	Phase I/II	Advanced solid tumors	Monotherapy olaparib, niraparib	Recruiting
ART4215	NCT04991480	Phase I	Advanced, metastatic solid tumors	Talazoparib, niraparib	Active, not recruiting
GSK4524101	NCT06077877	Phase I/II	Advanced solid tumors	Monotherapy	Active, not recruiting

*Source*: https://clinicaltrials.gov (August 26, 2024).

Novobiocin (NVB) is a coumarin antibiotic, derived from *Streptomyces*, that can bind the ATP‐binding site of DNA gyrase and inhibit ATP hydrolysis. It is a clear example of drug repurposing. In fact, it has already been tested in an oncological clinical trial, but not in preselected HR‐deficient tumors.^277^, ^278^ In vitro data suggested that NVB phenocopied the deletion of Polθ as inhibited TMJE activity in U2OS cells but not HR repair; in addition, RAD51 foci and γH2XA were increased in these treated cells. NBV specifically induced cell death in vitro *BRCA1*‐ and *BRCA2*‐knockout RPE1 cells, in TOV21G *FANCF*‐deficient cells but with no effect in the corresponding WT and/or complemented cells. Similar results were obtained after in vivo treatment with NVB 75 mg/kg twice a day 5 days a week for 4 weeks in mice transplanted with human and murine tumors. The treatment induced tumor regression/stabilization only in HR‐deficient background and had no effect in HR‐proficient tumors. The combination of NBV and olaparib was found not only to be synergistic, but also potentiate the activity of olaparib in vivo and to overcame acquired resistance to olaparib. In addition, the combination was well tolerated and had no toxic effects. The molecular basis of the NVB/olaparib combination's activity was elevated DSB end‐resection with accumulation of ssDNA non‐productive intermediates and non‐functional RAD51 loading, leading to cell death.^162^ A whole‐genomic CRISPR/Cas9 screening in two NSCLC cells *TP53*‐mutated identified loss of the TMEJ pathway as a determinant of DNA‐PKcs inhibitor (peposertib) sensitivity.^279^

NVB is currently under clinical investigation with a phase I trial to test the safety, side effects, and best dose of NVB in *BRCA*‐mutant and other DNA repair‐deficient solid tumors (NCT05687110).

ART558, ART812, and ART4215: ART558 was identified by high throughput screening in which more than 150,000 compounds were tested for their ability to inhibit the polymerase activity of the full‐length Polθ expressed in *Escherichia coli* and followed by structure‒activity relationship studies to optimize the hits.^117^ Mechanistic studies suggested that ART558 binds the allosteric binding site within the polymerase catalytic domain of Polθ and enhances its thermal stability when DNA is present.^117^ It inhibited the cellular TMEJ at sub‐micromolar concentrations without affecting NHEJ. The compound was much more effective in cells deficient in *BRCA2* than in WT cells, interfering with cell growth, colony formation, and inducing cell death through apoptosis, as already reported for siRNA against Polθ.^159^ The compound was also in synthetic lethality with loss of *BRCA1* as demonstrated by its extreme activity in *BRCA1‒/‒* syngeneic cells compared to WT cells and in cells with *BRCA1* pathogenic mutations (MDA‐MB‐436 breast cancer and COV362 ovarian cells).^117^ These same authors described a synthetic lethal interaction of ART558 with components of the 53BP1/Shieldin complex (SHLD2 and MAD2L2) in vitro and in vivo in *BRCA1* cells. There was no potentiating effect between ART558 and loss of *SHLD2* and *MAD2L2* in *BRCA1* WT cells. Studies on the mechanism of this interaction suggest that when both the 53BP1/Shieldin complex and BRCA1/2 functions are inactivated, Polθ is essential for repair of the resected ssDNA end derived from the action of the nuclease on DNA ends, as a consequence of the lack of Shieldin complex. The simultaneous inhibition of Polθ is responsible for the synthetic lethality. The Shieldin complex prevents resection at the DNA‐DBS and its loss has been described as a possible mechanism of PARPi resistance in *BRCA1* mutant cells,^280^ suggesting that ART558 could target cells that have become PARP inhibitor resistant due to the loss of Shieldin complex. Data on the ability of ART588 to radio‐sensitize human cells proficient for HR repair in normoxic and hypoxic conditions, frequently associated with in vivo tumor growth, was recently published.^167^ ART558 was not suitable for in vivo testing on account of its very high clearance in mouse microsomes (>1500 mL/min/mg) and its further chemical structure optimization led to the synthesis of ART812.^276^ ART812 pharmacokinetics and pharmacodynamic studies show good oral bioavailability and its ability to engage Polθ in vivo, as suggested by the increased MN formation in reticulocytes measured by flow cytometry.^276^ The same authors reported significant tumor growth inhibition of ART812 when given orally as monotherapy (100 mg/kg QD) in a rat *BRCA1*‒/‒ and *SHLD2*‒/‒ breast cancer xenograft model. ART899, a deuterated form of ART812, has reported to have greatly improved metabolic stability compared to ART588, with similar properties of Polθ inhibition in vitro, as regards potency and specificity. ART899 was able to radio‐sensitize human colon cancer cells by a factor of 4 and 5 when combined with fractionated IR (5 × 2 Gy) at non‐toxic concentration.^167^ No such effect was seen in cells deleted of Polθ, corroborating the tumor specificity of the compound. Lastly, this in vitro effect was also observed in vivo, where the combination of ART899 and fractionated irradiation (10 × 2 Gy) significantly improved tumor growth delay and mean survival of mice transplanted with HCT116 human cells compared to single treatments.^167^

Artios compounds have been used in clinical investigation. Specifically, ART4215 (undisclosed structure) is undergoing an open‐label phase I/IIa study designed to assess it ART4215 as an oral anticancer agent for monotherapy of patients with cancers with defects in DNA repair, and in combination with talazoparib or niraparib (NCT04991480). The study aims at defining the safety profile of 21‐day cycles. Phase b will enroll patients to be treated with ART4215 and talazoparib. A subsequent cohort will include patients already treated with PARPi. An expansion cohort will be patients with HER2‐negative *BRCA* breast cancers randomized 1:1 to receive either ART4215 with talazoparib or talazoparib alone. The study, involving multiple centers in the United States and Europe, started in September 2021 and the primary outcome results are expected by mid‐2025. ART6043 is Artios’ follow‐up Polθ inhibitor, which is also a selective, orally bioavailable, small molecule inhibitor of the Polθ polymerase domain (https://www.artios.com/pipeline/). The ART6043 phase I study (NCT05898399) started in 2Q2023 and will investigate the drug as monotherapy (part A1) and in combination with either olaparib (part A2) or talazoparib (part A3), in patients with advanced and metastatic solid malignancies with genetic lesions.

RP‐6685 has shown very potent (IC50 = 0.55 nM) and selective for Polθ, and completely inactive on other polymerases. The compound inhibited the polymerase activity of a full‐length Polθ produced in HEK293 cells, was inactive against its ATPase activity and induced a dose‐dependent decrease in TMEJ similar to that observed using siRNA targeting Polθ. In addition, it seems to bind the Polθ alloresteric site, similarly to compound ART558. As expected, RP‐6685 was more cytotoxic in *BRCA2*‒/‒ cells than *BRCA2* WT cells, although the IC50 was in the order of sub‐micromolar, with a substantial shift in potency compared to the nM range IC50 reported in the in vitro PicoGreen assay.^274^ The compound had a favorable pharmacokinetic profile when used orally and showed hints of antitumor activity in HCT116 *BRCA2*‒/‒ cells compared to HCT116 WT cells transplanted in nude mice. However, the antitumor activity was marginal and not sustained and paralleled the low potency of the compound in cells; experiments aimed at defining the magnitude of in vivo target engagement support its partial engagement as demonstrated by the increased micronuclei and γH2AX in cells derived from tumor xenografts treated in vivo with RP‐6685.

Many other Polθ inhibitors have been included in the pipeline of several biotech and pharmaceutical companies mainly focusing on synthetic lethality‐based drug discovery in homologous recombination deficiency tumors. GKS101, developed by IDEAYA Biosciences, is a Polθ helicase inhibitor developed in collaboration with GSK and a phase I trial is planned/running in combination with nirabarib in solid tumors with HR mutations (https://www.ideayabio.com/pipeline/).

## 5 CONCLUSION AND PROSPECTS

Targeting the DDR has clearly a therapeutic value in oncology as demonstrated by numerous preclinical and clinical evidences.^2^, ^3^ In the past decades more than 1000 compounds have been evaluated for their ability to inhibit ATM, ATR, Chk1, Wee1, and Polθ but only few have reached the clinical stage.^10^ The scarcity of high‐resolution structures of the target proteins, the large size of some of the proteins (i.e., ATM, ATR) and the requirement for addition proteins for their activity have rendered the design of structure‐based inhibitors challenging. The lack of high selectivity implies that these inhibitors could affect other family member (i.e., for ATM and ATR inhibitors, these include DNA‐PK, mTOR, and PIK3; for ChK1 inhibitors, these include Chk2, etc.) and could be responsible for some of the reported off‐target effects. Enhancing the selectivity will probably mitigate the off‐target toxicities. For example, azenosertib, a very selective Wee1 inhibitor, can be administered continuously with a much more manageable safety profile than adavosertib.^267^

Based on preclinical evidence, the ATM, ATR, Chk1, Wee1, and Polθ inhibitors have been mainly developed in two different settings: in monotherapy in tumors with specific defects in a synthetic lethal approach and in combination with cytotoxic or other DDR agents. In the former case, treatment with DDR inhibitors (DDRi) target the remaining DDR pathways, leaving normal tissues unaffected. Extensive preclinical research with high throughput screenings (CRISPR/Cas9 and/or chemical libraries) have led to the identification of these lethal interactions. While this has been to be particularly effective using PARPi in a HR‐deficient background, similar considerations could be done between ATR and the ATM/p53 pathway and between ATM and DNA‐PKs. A synthetic lethality between HR deficiency and Polθ has been corroborated by ample in vitro and in vivo experimental evidence. Precision medicine will have a very important role in matching specific genetic tumor alterations and the specific DDRi. While with the evolving molecular technologies is now possible to sequence easily tumor patients’ samples, the interpretation and deconvolution of the results could be problematic and there is the need to identify biomarkers of response to DDRi, that will certainly help in a better patients’ stratification, treating those patients with the best choice to respond and avoiding treatments in those who will not respond.

In combination therapy, all these DDRi have demonstrated to increase both the sensitivity to RT or radiomimetic drugs, classical chemotherapeutics (such as topoisomerase and gemcitabine) both in tumor cells and in normal cells, resulting in a higher incidence of hematological toxicities. These data suggest the need of more preclinical data to enhance the clinical benefit of these combinations possibly addressing smart dose regimens with the aim to reduce the myelotoxicity and other possible side effects. The clarification of the long‐term toxicities of these inhibitors is also very important and there are no data on the continuous, long‐term inhibition of most of the described inhibitors in adults. While the ongoing clinical studies will certainly characterize the safety profile of the different inhibitors in the time frame of each study, long‐term observation studies are needed to address this point. Preclinical emerging data support that combination among DDRi is feasible and could be of therapeutic value.^281^, ^282^, ^283^

There are two other important clinical settings where these drugs are currently being tested: in combination with immunotherapy and in the resistant setting. The interplay between immunity and DDR has been recently reviewed.^284^, ^285^ Genomic tumor alterations, DNA lesions induced by radio‐chemotherapy, DDRi, alone or in combination can cause accumulation of cytosolic DNA fragment able to activate the cGAS‐STING pathway and to trigger and modulate antitumor‐immune response.^285^ The results support the DDRi/immunotherapy combination that is now being tested in the clinical settings. Tumors will eventually develop resistance to therapy and for many anticancer agents, the mechanisms behind could be targeted by inhibition of DDR. For example, among the mechanism of PARPi resistance there are increased RS, perturbation of DNA replication that could be targeted by ATR/Chk1/Wee1 inhibitors.^103^, ^280^ Again, PARPi resistance in *BRCA*‐mutated tumors displays a TMEJ‐specific mutational signature, loss of *53BP1* is associated with PARPi resistance and this gene was found in synthetic lethality with Polθ^286^ suggesting a role in Polθ inhibitors in reverting PARPi resistance.

As discussed in this review, many ATM, ATR, Chk1, Wee1, and Polθ inhibitors have been recently developed and the ones showing promising results in the preclinical setting are now undergoing clinical trials in different settings, and for data are awaited most of them. However, it is imperative to focus on optimizing their administration (e.g., through superior formulation and better scheduling) to maximize clinical efficacy and reduce side effects.

## AUTHOR CONTRIBUTIONS

Guffanti Federica, Chiappa Michela, and Damia Giovanna conceptualized the manuscript and wrote, read, and critically revised the manuscript for intellectual content and all approved the final manuscript.

## CONFLICT OF INTEREST STATEMENT

The authors declare they have no conflicts of interest.

## ETHICS STATEMENT

Not applicable.

## Data Availability

Not applicable.
